# Acute Response of Different High-Intensity Interval Training Protocols on Cardiac Auto-Regulation Using Wearable Device

**DOI:** 10.3390/s24144758

**Published:** 2024-07-22

**Authors:** Myong-Won Seo

**Affiliations:** Department of Sport and Leisure Studies, College of Physical Education, Keimyung University, Daegu 42601, Republic of Korea; myongwonseo@kmu.ac.kr; Tel.: +82-53-580-5257

**Keywords:** optimal HIIT, HRV, ba-PWV, frequency domain, ankle–brachial index

## Abstract

The purpose of this study was to compare different high-intensity interval training (HIIT) protocols with different lengths of work and rest times for a single session (all three had identical work-to-rest ratios and exercise intensities) for cardiac auto-regulation using a wearable device. With a randomized counter-balanced crossover, 13 physically active young male adults (age: 19.4 years, BMI: 21.9 kg/m^2^) were included. The HIIT included a warm-up of at least 5 min and three protocols of 10 s/50 s (20 sets), 20 s/100 s (10 sets), and 40 s/200 s (5 sets), with intensities ranging from 115 to 130% Watt_max_. Cardiac auto-regulation was measured using a non-invasive method and a wearable device, including HRV and vascular function. Immediately after the HIIT session, the 40 s/200 s protocol produced the most intense stimulation in R-R interval (Δ-33.5%), ln low-frequency domain (Δ-42.6%), ln high-frequency domain (Δ-73.4%), and ln LF/HF ratio (Δ416.7%, all *p* < 0.05) compared to other protocols of 10 s/50 s and 20 s/100 s. The post-exercise hypotension in the bilateral ankle area was observed in the 40 s/200 s protocol only at 5 min after HIIT (right: Δ-12.2%, left: Δ-12.6%, all *p* < 0.05). This study confirmed that a longer work time might be more effective in stimulating cardiac auto-regulation using a wearable device, despite identical work-to-rest ratios and exercise intensity. Additional studies with 24 h measurements of cardiac autoregulation using wearable devices in response to various HIIT protocols are warranted.

## 1. Introduction

Cardiovascular auto-regulation provides homeostasis to the human body through blood flow regulation, despite changes in perfusion/pressure [1,2]. The autonomic nervous system (ANS) consists of the sympathetic nervous system (SNS) and the parasympathetic nervous system (PNS), which regulate involuntary physiological responses, including heart rate variability (HRV) and vascular function [3,4]. The SNS and PNS are composed of two nerve fibers (afferent and efferent neurons) that transmit sensory input and motor output signals to the central nervous system for sensory integration [5,6]. The SNS is activated by moderate-to-vigorous physical activity, stress, or dangerous situations (known as fight-or-flight reactions), increasing heart rate, blood pressure, and vasoconstriction [7,8]. Conversely, the PNS is called “resting and digesting”, which is associated with the restoration and recovery processes of the human body by modulating heart rate and blood pressure [9]. ANS dysfunction is increasingly recognized as potentially exacerbating glucose regulation and cardiac conditions that can lead to cardiometabolic diseases [10,11].

The HRV is a non-invasive tool that reflects the ANS and independently predicts cardiometabolic diseases [12]. HRV usually consists of two domains: time and frequency. For the time domain, the interval between heartbeats (inter-beat interval [IBI]) and the time between R waves of the QRS complex in electrocardiography (R-R interval) within the recording times are analyzed [13]. Additionally, the time domain is used to quantify the amount of HRV, eliciting SDNN (standard deviation of normal-normal beats), rMSSD (root mean square of the successive differences), pNN50 (proportion of NN50 divided by the total number of NN intervals), etc. For the frequency domain, the power spectral densities of the R-R interval time series are calculated using a fast Fourier transformation or autoregressive algorithm. Frequency domains are commonly used for sympathetic activity in the low-frequency domain (LF, 0.04–0.15 Hz), parasympathetic activity in the high-frequency domain (HF, 0.15–0.40 Hz), and sympathovagal balance in the LF/HF ratio [14]. A widely used non-invasive method to measure blood pressure is using a pressurized cuff around the brachial and ankle. Blood pressure is associated with cardiovascular disease risk factors [15]. In addition, brachial–ankle pulse wave velocity (ba-PWV) is the predicted arterial stiffness [16], which is assessed using the difference between brachial and tibial artery length divided by the time difference between the starting points of systolic rise in brachial and tibial pressure waveforms [17]. Importantly, previous studies have demonstrated that measurements of HRV, brachial and ankle blood pressure, ankle–brachial index, and ba-PWV can provide valuable information for identifying participants with a high risk of cardiometabolic diseases [18,19,20].

High-intensity interval training (HIIT), which consists of repeated bouts of high-intensity exercise interspersed with a period of recovery, has been demonstrated as a time-efficient strategy for improving cardiovascular health in healthy and unhealthy populations [21,22,23]. Previous studies have reported that HIIT stimulates intense cardiac structure and function [24,25], along with repairing ANS function and mitigating cardiovascular disease risk factors [26,27,28]. Besnier et al. showed that HIIT was significantly superior to traditional aerobic exercise (moderate-intensity continuous training, MICT) in cardiac auto-regulation, including ANS and cardiovascular function [29]. Nevertheless, it is unclear what the optimal HIIT protocol is for cardio-autoregulation.

Cardiac auto-regulation is important for the brain and heart and maintains homeostasis for physical function [30]. However, evidence-based literature regarding the response to a single session of different protocols on HRV using wearable devices and vascular function is lacking. Given that long-term exercise strategies should be carefully designed, acute physiological responses during single sessions should be preferentially identified. The present study aimed to compare different HIIT protocols by utilizing different work and rest times for each single HIIT session with identical work-to-rest ratio (1:5) and intensity (115–130 Watt_max_ [W_max_]) and to analyze the effects on HRV using a wearable device and assessing vascular function. In addition, this study might provide insights into the mechanisms underlying the ANS and vascular function after HIIT.

## 2. Materials and Methods

### 2.1. Participants

A power analysis using G*Power program 3.1.9.2 (Dusseldorf, Germany) was used to determine the sample size required to detect within-between factors for a repeated-measures analysis of variance (ANOVA). With an estimated power of 0.95 and an alpha of 0.05, a total sample of 12 was required to detect a partial eta squared of 0.33. Thirteen young male adults volunteered to participate in the present study (age: 19.4 ± 0.3 years, height: 172.2 ± 1.58 cm, body weight: 64.9 ± 0.4 kg, body mass index (BMI): 21.9 ± 0.5 kg/m^2^, fat mass: 8.6 ± 0.8 kg, fat free mass: 53.0 ± 1.2 kg, percent body fat: 11.7 ± 0.6%). Participants were required to meet the following inclusion criteria: (a) age between 19 and 25 years, (b) normal weight (18.5 kg/m^2^ ≤ BMI < 23 kg/m^2^), (c) no history of any disease, relevant medication, and neuro-skeletal muscular injury, and (e) physically active lifestyle. Physical activity (PA) was measured using the ActiGraph GT9X accelerometer (ActiGraph Corp., Pensacola, FL, USA), which was continuously worn on the non-dominant wrist for seven consecutive days. We applied cut-off points for moderate and vigorous PA by Montoye et al. [31]. Physical inactivity was defined as less than 150 min/week of moderate-to-vigorous PA (MVPA). The purpose, design, procedure, and potential benefits and risks were fully explained to the participants, and written informed consent was obtained from all participants.

### 2.2. Experimental Design

This study compared the acute response of HRV and vascular function after HIIT in healthy young adults. In a randomized crossover counter-balanced design, 13 physically active young adult participants performed three HIIT protocols. Participants signed an informed consent form on the first visit, and their physical characteristics and body composition were measured. Then, the maximal graded exercise test (GXT) was performed on a cycle ergometer (Monark 828E, Varverg, Sweden) to assess the exercise intensities. Briefly, the GXT was assessed after a 5 min warm-up at 60 rpm/0.5 kilopounds (kp), increasing to 1 kp to begin the test. Subsequently, every 2 min, the resistance was increased by 0.5 kp, with a pedaling cadence rate of 60 rpm. The GXT was discontinued when three or more of the following criteria were met: (1) participants requested to stop; (2) the estimated heart rate exceeded 90% of the maximum (220-age), (3) the rate of perceived exertion (RPE) equaled or exceeded 17, or (4) participants could no longer maintain a pedaling cadence of 60 rpm (exhaustion). Before the second visit, all participants were provided with an accelerometer to be worn on the non-dominant wrist to obtain physical activity (PA) data for 24 h over seven days of free-living PA. HRV and vascular function were measured according to acute response following various HIIT protocols at the second and subsequent visits. Participants were instructed not to drink caffeine or alcohol for at least 24 h before the examination. HRV was assessed at resting baseline and immediately after HIIT, as well as 15, 30, 45, and 60 min after HIIT. The vascular function test was performed at resting baseline and 5, 20, 50, and 65 min after HIIT. For recovery monitoring, we did not perform a vascular function test immediately after HIIT because of the possibility of the vascular function test affecting HRV.

### 2.3. Anthropometric and Body Composition

Body height and weight were measured with participants wearing light clothing without shoes, to the nearest 0.1 cm using a standard stadiometer (aluminum anthropometer, Samhwa instruments, Seoul, Republic of Korea) and an electronic scale (150A CAS, Seoul, South Korea) to the nearest 0.01 kg. BMI was calculated as body weight (kg) divided by height in meters square (m^2^). Body composition parameters, including fat mass, body fat percentage, and lean body mass, were assessed using dual X-ray absorptiometry (DEXA; QDR-4500W, Marlborough, MA, USA). The coefficient of variance of DEXA measurements was 1.5% or less. The same technician performed all scans, and the intraclass correlation coefficient was 0.99.

### 2.4. Heart Rate Variability (HRV)

HRV was measured using the Active Cardio monitor (Actiwave cardio, CamNtech, UK) [32]. The device is a commonly used portable electrocardiogram (ECG) waveform recorder for HRV research, and its validation has been described in previous studies [33,34,35]. Active Cardio is an extremely small (1 cm thickness, 3.2 cm diameter, 10.3 g) two-electrode device that can measure ECG using a soft elasticated chest belt. The sample rate for the ECG signals was set to 1204 Hz, with a 10-bit resolution. HRV was measured in the supine position without any movement in a quiet and dark room. Baseline HRV data were assessed for 15 min, of which the first 10 min were deleted to avoid collecting potentially corrupt data and the last 5 min were selected for analysis according to autonomic nervous activity, according to the Task Force of the European Society of Cardiology and North American Society of Pacing and Electrophysiology guidelines [36]. For instance, HRV data were analyzed in short-term periods of 5 min using the HRV analysis software program. Data analysis was performed using the Kubios HRV Premium software (Version 3.5.0, Kuopio, Finland). HRV variables include time domain (R-R intervals, SDNN, rMSSD, pNN50) and frequency domain (ln LF: 0.04–0.15 Hz, ln HF: 0.15–0.45 Hz, ln LF/HF ratio: LF divided by HF) analyses.

### 2.5. Vascular Function

Vascular function was measured using a non-invasive vascular monitoring tool (VP-1000; Omron Healthcare, Kyoto, Japan). This portable device is a validated pulse wave velocity tool [37] used to measure arterial stiffness as a cardiovascular disease risk predictor. To assess vascular function, the participants were placed in a supine position on a comfortable bed, and pneumatic pressure cuffs were wrapped tightly around the bilateral brachial and ankle of the participants. Vascular function included bilateral brachial and ankle blood pressure variables (systolic blood pressure [SBP], diastolic blood pressure [DBP]), ankle–brachial index (ABI), and bilateral ba-PWV. This tool simultaneously assessed the pulse wave velocity, phonocardiogram, and electrocardiogram; two measurements were taken for each evaluation, and the average of the two measurements was used.

### 2.6. HIIT Protocol

The HIIT protocols were designed by a certified strength and conditioning specialist (CSCS). The participants performed the three protocols with a seven-day interval over 21 days using a Monark anaerobic wingate ergometer cycle (Monark 894E, Sweden). The HIIT included a warm-up of at least 5 min without resistance and 3 protocols with different lengths of work and rest time: 10 s/50 s, 20 s/100 s, and 40 s/200 s, with identical work-to-rest ratios and an intensity ranging from 115 to 130%W_max_. During the HIIT, exercise intensity was monitored using a software program (Monark Anaerobic Wingate Software 3.0, Varverg, Sweden). All HIIT protocols were equal in total volume (20 min), work-to-rest ratio (1:5), total work time (200 s), and total rest time (1000 s). However, the number of sets for each protocol was different (10 s/50 s = 20 set, 20 s/100 s = 10 set, 40 s/50 s = 5 sets). The recovery phase for each protocol was set to 60 rpm and pedaled without resistance. Additionally, the CSCS encouraged the exercise to target the 115–130 W_max_ intensity zone, and the RPE was obtained from the participants immediately after each set.

### 2.7. Statistical Analysis

Data were analyzed using IBM SPSS (version 28 for Mac; IBM Corp., Armonk, NY, USA). All data are expressed as the mean and standard error of the mean. Repeated-measures ANOVAs were used to determine the significant interaction effect for the group by time. In addition, one-way ANOVA was used to compare between and within groups, with Bonferroni correction for multiple tests. The effect sizes (ESs) were calculated as partial eta-squared values. The statistical significance level was set at *p* < 0.05.

## 3. Results

Table 1 presents the exercise volume, peak heart rate, and RPE during HIIT. There were no significant differences in the mean and relative mean power outputs. However, there were significant differences in peak heart rate (HR_peak_) and RPE. Among the three protocols, there were progressive increases in the HR_peak_ from 10 s/50 s to 20 s/100 s to 40 s/200 s protocols (*p* < 0.001). In addition, the 40 s/200 s protocol had a higher value of RPE than the 10 s/50 s and 20 s/100 s protocols (*p* < 0.001).

**Figure 1:**
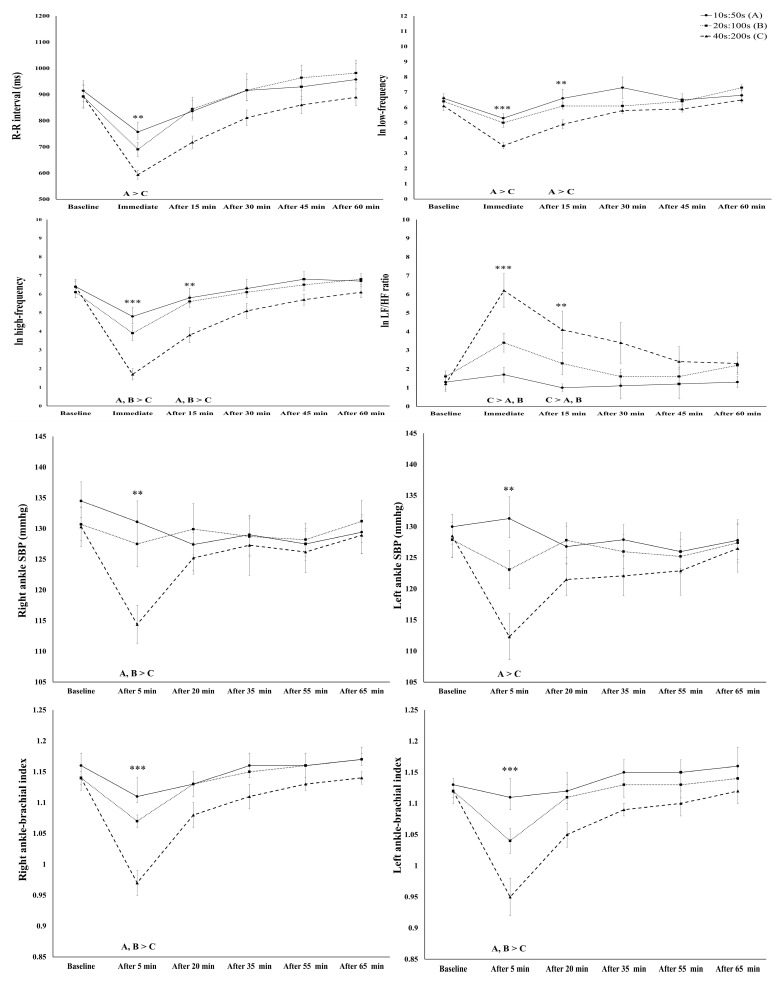
Acute cardiac auto-regulation responses to different HIIT protocol. Note—SBP: systolic blood pressure, DBP: diastolic blood pressure, * Significant difference effect among the groups, ** *p* < 0.01, *** *p* < 0.001.

Our repeated-measures ANOVA exhibited a significant interaction effect between groups by time in heart rate (HR) and R-R interval (F = 2.24, *p* < 0.05, $\eta_{p}^{2}$: 0.11). Although RMSSD, SDNN, and pNN50 showed a significant main effect of time on the acute response to HIIT (*p* < 0.001), there was no significant interaction effect between groups over time (Table 2). HRs measured at time points immediately after and 15 min after HIIT were higher in the 40 s/200 s protocol than the 10 s/50 s and 20 s/100 s protocols (*p* < 0.01), and in the 40 s/200 s protocol, they were higher than the 10 s/50 s protocol at 30 min after HIIT (*p* < 0.05). The R-R interval measured immediately after HIIT was higher in the 10 s/50 s protocol than the 40 s/200 s protocol (*p* < 0.01). There was a significant interaction effect for groups by time in ln LF (F = 2.34, *p* < 0.05, $\eta_{p}^{2}$: 0.12), ln HF (F = 5.60, *p* < 0.01, $\eta_{p}^{2}$: 0.24), and ln LH/HF ratios (F = 4.13, *p* < 0.01, $\eta_{p}^{2}$: 0.20) (Figure 1). Especially, the ln LF of the 10 s/50 s protocol was higher than that of the 40 s/200 s protocol at the time points immediately after (*p* < 0.001) and 15 min after HIIT (*p* < 0.01). The ln HF in the 10 s/50 s and 20 s/100 s protocols was higher immediately after (*p* < 0.001) and 15 min after HIIT (*p* < 0.01) than in the 40 s/200 s protocol. In addition, the ln LF/HF ratio of the 40 s/200 s protocol was higher than that of the 10 s/50 s and 20 s/100 s protocols at the time points immediately after (*p* < 0.001) and 15 min after HIIT (*p* < 0.01). In the bilateral brachial SBP, no significant interaction effect was observed between the groups by time. However, there was a significant interaction effect between the groups by time for bilateral ankle SBP (right, F = 2.99, *p* < 0.01, $\eta_{p}^{2}$: 0.14; left; F = 4.18, *p* < 0.001, $\eta_{p}^{2}$: 0.19). However, right and left ankle SBP in the 10 s/50 s and 20 s/100 s protocols was higher 5 min after HIIT compared with the 40 s/200 s protocol (*p* < 0.01) (Figure 1). For bilateral ba-PWV, despite a significant main effect of time, there was no significant interaction effect for the groups by time. The bilateral ABI showed a significant interaction effect between the groups by time (right, F = 3.02, *p* < 0.01, $\eta_{p}^{2}$: 0.14; left, F = 4.73, *p* < 0.001, $\eta_{p}^{2}$: 0.21). For 5 min after HIIT, the right ABI was higher in the 10 s/50 s protocol than in the 40 s/200 s protocol, and the left ABI was higher in the 10 s/50 s and 20 s/100 s protocols than in the 40 s/200 s protocol (all *p* < 0.001). See Table 3.

## 4. Discussion

We, for the first time, provided evidence of acute cardiac auto-regulation response—using a wearable device—to various HIIT protocols in a single session in physically active young adults. Specifically, we demonstrated that (1) the 10 s/20 s, 20 s/100 s, and 40 s/200 s protocols produced significant changes in HRV time domain and frequency parameters after a single session of HIIT; (2) the 40 s/200 s protocol produced the most intense stimulation in heart rate, R-R interval, ln LF, ln HF, and ln LF/HF ratio compared to the 10 s/20 s and 20 s/100 s protocols; (3) both the SNS and PNS were activated more in the 40 s/200 s protocol; (4) although there was no significant interaction effect for groups by time for bilateral brachial SBP, post-exercise hypotension in the bilateral ankle area was shown in the 40 s/200 s protocol only; and (5) bilateral ABI decreased from the normal range to approximately 0.97 in the 40 s/200 s protocol after HIIT.

HRV has been widely used as a surrogate marker of cardiac autonomic function, calculated using electrocardiographic waveforms, reflecting both SNS and PNS activity [38]. Hence, HRV measurement is needed to identify whether ANS function is impaired in physiological and pathophysiological health statuses [8]. In the present study, PNS activity seemed to be temporarily downregulated until at least 30 min after HIIT, which is consistent with the results of previous studies [39]. Exercise-induced hypotension causes a decrease in afferent input from baroreceptor signals [40,41] and leads to an increase in SNS activity [42]. Additionally, the SNS is highly stimulated by the metaboreflex and neurotransmitter secreted by nerves, and PNS re-activity after exercise is significantly associated with principal neurotransmitters, such as epinephrine [43,44]. This study found that heart rate, R-R interval, ln LF, ln HF, and ln LF/HF ratio in acute response to HIIT varied with different work and rest times, despite identical work-to-rest ratios and exercise intensity. In addition, all HRV time domains using wearable devices changed in all three protocols after HIIT, but they all recovered to baseline before 60 min. Given that exercise intensity is the most important factor in increasing HRV [45,46], we expected no distinct difference between the protocols and the recovery phase. One study by Cipryan compared the cardiac auto-regulation response to single HIIT sessions at different fitness levels [47]. This study showed that fitness level was positively associated with the fast recovery of PNS activity following HIIT. Furthermore, Schaun et al. compared different exercise modes with HIIT in the cardiac auto-regulation response [45]. They demonstrated that responses to HRV were similar between training modes (cycling vs. whole-body). In the present study, a longer work time (40 s) was associated with increased heart rate, R-R interval, ln LF, ln HF, and ln LF/HF ratio than a shorter work time (10 s and 20 s), regardless of the equal work-to-rest ratios and exercise intensity. For the RPE, the 40 s/200 s protocol was higher than the 10 s/20 s and 20 s/100 s protocols in the present study. Therefore, RPE and physical fitness levels may have influenced the ANS during the recovery process after HIIT.

The blood pressure and ba-PWV were associated with independent risk factors of cardiovascular disease [48,49], which reflects arterial stiffness [50]. Arterial stiffness alterations are affected by blood flow dynamics, which are controlled by cardiac auto-regulation [51,52]. This study showed that the 40 s/200 s protocol was lower than the 10 s/50 s and 20 s/100 s protocols at the time points of 5 min after HIIT in bilateral brachial SBP and ABI and then recovered to baseline before 20 min. Eduardo et al. reported that HIIT was significantly associated with longer post-exercise hypotension compared to traditional aerobic exercise but did not change DBP and arterial compliance in middle-aged and older hypertensive women with increased arterial stiffness [53]. In addition, Ramirez-Jimenez et al. demonstrated that the blood pressure response to a single HIIT session is more strongly stimulated compared to isocaloric MICT [54]. Post-HIIT produces flow-mediated vasodilation due to increased shear stress by plasma viscosity status [55], which reflects central sympathetic control and endothelial dilatory mechanisms. Collectively, we speculated that for the 40 s/200 s protocol at the time points between immediately after and 15 min after HIIT, peripheral blood vessels were more dilated, with decreased circulating catecholamines and sympathetic nervous activity compared to the 10 s/50 s and 20 s/100 s protocols. Therefore, our results support previous findings that a longer exercise time is a more potent regulator of the acute response to vascular functions after HIIT despite equal exercise intensity.

Our study has strengths and limitations that should be considered when interpreting its results. The strengths are summarized as follows: (1) the study was designed with a randomized crossover counter-balance; hence, all the research processes were supervised by the CSCS from this research project group; (2) it is a first-time investigation to compare different HIIT protocols by utilizing different work and rest times for single HIIT sessions with identical work-to-rest ratio and exercise intensity and analyzing the effects on HRV and vascular function; and (3) the study used non-invasive methods for HRV and vascular functions (measured using external electrodes and oscillometric methods). Nevertheless, this study has several limitations, including the small sample size and lack of the time point of measurement (HRV: 60 min or less, vascular function 65 min or less). In addition, the vascular function was not assessed immediately after HIIT because inflating the blood pressure cuff can affect HRV parameter results.

By knowing which HIIT protocol is more efficacious in improving cardiac auto-regulation, exercise physiologists, researchers, and clinicians can properly prescribe exercise and possibly prevent disease progression. The findings of this study demonstrated that a gradual increase in work time might be more effective in stimulating cardiac auto-regulation, despite identical work-to-rest ratios and exercise intensities. This study suggests that if more stimulation of heart function is desired through HIIT, a longer work time seems to be a better strategy. Nevertheless, we performed a pilot test to examine five sets of 40 s exercise time at 130–150% W_max_, but the participants did not complete the session due to exhaustion. Given the abovementioned information, exercise duration, recovery times, and exercise time should be carefully considered when designing HIIT protocols.

## 5. Conclusions

This study confirmed that a longer work time in 115–130 W_max_ and 1:5 ratio (immediately after the HIIT, 40 s/200 s; R-R interval [Δ-33.5%], ln low-frequency domain [Δ-42.6%], ln high-frequency domain [Δ-73.4%], and ln LF/HF ratio [Δ416.7]; 5 min after HIIT, 40 s:200 s; bilateral ankle area (right: Δ-12.2%, left: Δ-12.6%)) might be more effective and sensitive for stimulating sympathovagal balance and endothelium-mediated dilation and lead to favorable cardiac-auto regulation, despite identical work-to-rest ratios and exercise intensities. Additional studies with 24 hr measurement of HRV and vascular function in response to various HIIT protocols are warranted and should be conducted in patients with cardiovascular disease.

## Figures and Tables

**Table 1:** Comparison of exercise power output, peak heart rate, and rate of perceived exertion during HIIT.

	10 s:50 s	20 s:100 s	40 s:200 s	*p* Value
Mean power output (Watt)	285.9 ± 9.2	290.6 ± 8.8	293.1 ± 8.2	0.842
Relative mean power output (Watt/kg)	4.4 ± 0.1	4.5 ± 0.1	4.5 ± 0.1	0.794
Peak heart rate (bpm·min^−1^)	139.0 ± 4.4 a	152.3 ± 3.8 b	174.0 ± 2.6 c	0.001
Rate of perceived exertion	11.9 ± 0.8 a	13.5 ± 0.7 a	16.9 ± 0.4 b	0.001

Same letters are not significantly different from each protocol.

**Table 2:** Acute heart rate variability response to high-intensity interval training among the various protocol.

Variables	Protocol	Baseline	Immediate	After 15 min	After 30 min	After 45 min	After 60 min	$F\;\mathrm{Value}\;(\eta_{p}^{2})$		
								Group	Time	G × T
Heart rate, bpm·min^−1^	10 s:50 s	67.0 ± 2.7	81.6 ± 4.0 ^a^	69.7 ± 2.9 ^a^	63.4 ± 2.2 ^a^	67.1 ± 5.4	62.8 ± 3.7	3.31 * (0.16)	74.71 *** (0.68)	4.63 *** (0.20)
	20 s:100 s	69.8 ± 3.1	88.5 ± 4.0 ^a^	72.5 ± 3.0 ^a^	67.0 ± 3.0 ^ab^	64.3 ± 3.5	62.3 ± 2.7			
	40 s:200 s	66.8 ± 3.4	102.1 ± 3.0 ^b^	84.7 ± 2.7 ^b^	75.1 ± 2.7 ^b^	70.9 ± 2.5	68.5 ± 2.4			
rMSSD, ms	10 s:50 s	55.5 ± 11.1	28.0 ± 6.7	49.7 ± 11.4	62.8 ± 17.0	65.2 ± 20.3	70.7 ± 21.1	2.12 (0.11)	18.49 *** (0.34)	1.56 (0.08)
	20 s:100 s	45.6 ± 8.8	18.3 ± 5.5	31.5 ± 3.8	40.6 ± 5.0	48.8 ± 6.5	58.1 ± 7.0			
	40 s:200 s	55.7 ± 13.2	5.5 ± 0.8	14.8 ± 3.2	25.7 ± 4.8	34.1 ± 5.8	40.2 ± 6.6			
SDNN, ms	10 s:50 s	47.0 ± 7.3	25.3 ± 4.1	42.1 ± 7.9	50.5 ± 10.6	55.7 ± 13.0	57.2 ± 13.4	3.05 (0.15)	24.54 *** (0.41)	0.68 (0.04)
	20 s:100 s	40.6 ± 5.7	21.2 ± 4.9	32.6 ± 2.8	37.6 ± 4.0	42.5 ± 4.0	57.7 ± 4.9			
	40 s:200 s	35.4 ± 4.0	8.2 ± 1.0	18.2 ± 2.9	27.5 ± 3.4	33.8 ± 3.8	41.5 ± 4.4			
pNN50, %	10 s:50 s	28.6 ± 7.5	13.9 ± 5.9	34.6 ± 12.2	40.6 ± 12.7	42.8 ± 14.7	43.6 ± 12.3	2.84 (0.14)	12.91 *** (0.26)	1.81 (0.09)
	20 s:100 s	18.7 ± 5.3	3.6 ± 2.4	14.1 ± 3.7	23.1 ± 4.9	30.6 ± 6.0	33.8 ± 5.3			
	40 s:200 s	27.4 ± 7.3	0.0 ± 0.0	3.3 ± 2.1	9.7 ± 4.4	16.4 ± 5.6	20.3 ± 6.5			

Note—rMSSD: root mean square of successive differences between normal heartbeats, SDNN: standard deviation of NN intervals, pNN50: percent of consecutive heartbeats with a difference of at least 50 ms, * Significant interaction and main effect, * *p* < 0.05, *** *p* < 0.001. Same letters are not significantly different from each protocol.

**Table 3:** Acute brachial–ankle pulse wave velocity to high-intensity interval training among the various protocols.

Variables		Protocol	Baseline	Immediate	After 15 min	After 30 min	After 45 min	After 60 min	$F\;\mathrm{Value}\;(\eta_{p}^{2})$		
									Group	Time	G × T
Right- Brachial (mmHg)		10 s:50 s	114.1 ± 2.5	116.9 ± 1.9	112.7 ± 1.7	110.2 ± 1.3	109.5 ± 1.8	110.0 ± 2.1	0.12 (0.89)	18.87 *** (0.34)	0.30 (0.02)
	SBP	20 s:100 s	114.4 ± 2.2	117.8 ± 2.7	114.4 ± 2.3	111.2 ± 2.3	110.2 ± 2.2	111.2 ± 2.7			
		40 s:200 s	114.4 ± 2.5	116.7 ± 1.8	115.1 ± 1.5	111.6 ± 1.8	110.9 ± 2.3	112.4 ± 2.3			
	DBP	10 s:50 s	60.1 ± 2.0	54.5 ± 1.2	59.5 ± 1.4	58.2 ± 1.3	58.9 ± 1.6	58.8 ± 1.8	0.36 (0.02)	7.48 (0.17)	0.55 (0.30)
		20 s:100 s	61.8 ± 1.8	56.4 ± 1.2	62.9 ± 2.9	60.6 ± 2.5	56.8 ± 1.6	59.8 ± 2.0			
		40 s:200 s	62.9 ± 1.8	54.8 ± 2.8	61.7 ± 1.3	60.0 ± 2.1	59.5 ± 2.1	60.2 ± 2.0			
Left- Brachial (mmHg)		10 s:50 s	113.8 ± 2.3	115.9 ± 1.7	111.2 ± 1.6	109.2 ±1.6	107.7 ± 2.0	110.5 ± 3.3	0.06 (0.00)	15.96 *** (0.31)	0.63 (0.03)
	SBP	20 s:100 s	113.2 ± 2.2	116.9 ± 3.1	113.2 ± 2.6	109.2 ± 2.4	109.4 ± 2.4	110.3 ± 2.6			
		40 s:200 s	111.9 ± 2.7	116.2 ± 2.4	113.6 ± 1.3	110.8 ± 2.3	109.8 ± 2.5	111.8 ± 2.5			
	DBP	10 s:50 s	60.5 ± 1.9	61.8 ± 1.7	59.5 ± 1.5	59.2 ± 1.6	58.0 ± 1.7	59.5 ± 2.1	0.36 (0.70)	4.70 *** (0.12)	0.61 (0.03)
		20 s:100 s	61.9 ± 1.7	62.2 ± 2.3	58.7 ± 1.9	60.5 ± 2.5	56.9 ± 1.8	60.2 ± 2.1			
		40 s:200 s	60.6 ± 2.1	61.9 ± 2.1	61.3 ± 1.6	59.7 ± 2.1	59.1 ± 2.5	60.0 ± 2.5			
Right- ankle (mmHg)	DBP	10 s:50 s	63.9 ± 1.8	63.6 ± 1.4	62.9 ± 1.4	63.4 ± 1.2	64.8 ± 2.5	64.0 ± 2.0	0.55 (0.03)	1.23 (0.03)	1.07 (0.06)
		20 s:100 s	64.8 ± 2.5	64.4 ± 2.4	62.8 ± 2.1	62.1 ± 3.1	61.6 ± 2.4	64.7 ± 2.6			
		40 s:200 s	62.5 ± 1.6	58.7 ± 2.3	62.9 ± 2.3	60.9 ± 2.8	58.7 ± 3.4	62.5 ± 3.1			
Left- ankle (mmHg)		10 s:50 s	63.2 ± 1.7	64.1 ± 1.6	63.9 ± 1.5	64.3 ± 1.5	64.7 ± 2.2	64.9 ± 2.1	0.86 (0.05)	1.46 (0.04)	1.24 (0.07)
	DBP	20 s:100 s	63.6 ± 2.2	62.8 ± 2.2	61.9 ± 2.2	62.1 ± 3.5	60.2 ± 2.6	63.6 ± 1.9			
		40 s:200 s	63.9 ± 2.1	57.4 ± 2.4	60.9 ± 2.4	59.9 ± 3.1	59.1 ± 3.2	61.0 ± 3.2			
ba-PWV (cm·s^−1^)	Right	10 s:50 s	1050.5 ± 33.4	1055.5 ± 36.8	1031.8 ± 34.9	1053.5 ± 29.8	1060.9 ± 37.5	1092.1 ± 34.1	0.49 (0.03)	6.49 *** (0.15)	1.86 (0.09)
		20 s:100 s	1059.4 ± 38.0	1041.0 ± 40.9	1035.3 ± 37.0	1043.3 ± 32.7	1037.2 ± 46.7	1088.3 ± 33.0			
		40 s:200 s	1053.0 ± 47.5	954.9 ± 36.9	999.2 ± 32.0	997.9 ± 30.9	1037.0 ± 32.1	1040.6 ± 32.8			
	Left	10 s:50 s	1059.9 ± 32.3	1044.0 ± 34.7	1021.6 ± 30.5	1040.3 ± 26.6	1053.4 ± 34.1	1092.1 ± 34.1	0.31 (0.02)	7.06 *** (0.16)	1.51 (0.08)
		20 s:100 s	1077.4 ± 38.2	1046.4 ± 38.1	1039.5 ± 36.9	1033.1 ± 28.7	1033.1 ± 45.4	1088.3 ± 33.0			
		40 s:200 s	1070.2 ± 43.5	964.9 ± 39.8	1016.9 ± 29.0	1010.5 ± 29.1	1030.3 ± 27.9	1040.6 ± 32.8			

Note—SBP: systolic blood pressure, DBP: diastolic blood pressure, ba-PWV: brachial–ankle pulse wave velocity. * Significant main effect, *** *p* < 0.001. There were no differences among the protocols for any of the variable.

## Data Availability

The data that support the findings of this study are available from the corresponding author upon reasonable request.
